# Acne treatment: research progress and new perspectives

**DOI:** 10.3389/fmed.2024.1425675

**Published:** 2024-07-10

**Authors:** Yuwei Li, Xinhong Hu, Gaohong Dong, Xiaoxia Wang, Tao Liu

**Affiliations:** Department of Dermatology, Tangdu Hospital, Air Force Military Medical University, Xi’an, China

**Keywords:** acne, research progress, antimicrobial resistance, novel antibiotics, probiotics

## Abstract

Acne is a chronic inflammatory skin disease that primarily affects adolescents and is attributed to various factors, including hormonal changes, genetic predisposition, and environmental influences. It typically manifests in areas rich in sebaceous glands such as the face, chest, and back. Symptoms of acne can range from mild to severe and may present as pimples, pustules, nodules, cysts, and scarring. The appearance of acne can significantly impact both the physical and mental well-being of patients, potentially leading to feelings of anxiety, depression, and social withdrawal. The pathogenesis of acne is multifaceted involving genetic predisposition as well as environmental factors such as hormonal imbalances, inflammation, abnormal follicular sebaceous unit keratinization, proliferation of follicular microorganisms like *Propionibacterium acnes*, increased sebum production, and dietary influences. Traditional treatment methods for acne include topical drug therapy, oral drug therapy, photoelectric therapy, and chemical peeling. With ongoing research into the pathogenesis of acne, treatment methods are rapidly evolving with novel antibiotics, probiotics, biological agents, topical anti-androgen drugs, topical vitamin A acid metabolism blockers, antimicrobial peptides, immunotherapy, micro-needling, and micro-needling patches. This article aims to provide a comprehensive review of recent advancements in acne treatment.

## 1 Introduction

Acne is a chronic inflammatory skin disease that primarily affects the follicular sebaceous units. The highest incidence of acne occurs between the ages of 15 and 20. The prevalence of acne is relatively low among pre-adolescent children, and while it decreases after puberty. However, it’s worth noting that a significant proportion of adults still suffer from acne (1). The pathogenesis of acne is complex and multifactorial, involving genetic predisposition, metabolism, hormone levels, environment, diet, immunity, etc. Common clinical manifestations of acne include pimples, papules, pustules, nodules, cysts, and scars, which seriously affect the physical and mental health of patients, and even some patients have anxiety, depression, and even suicidal tendencies (2, 3). The primary goal of acne treatment is to effectively manage and address current acne lesions, aim to prevent the formation of permanent scars, shorten the duration of the condition, and minimize the frequency of recurrence. Traditional treatment methods include topical and systemic use of antibiotics and retinoids, oral anti-androgens, phototherapy, and chemical peeling, depending on the individual’s specific case, taking into account factors such as the type and severity of acne lesions, overall health status, and any potential side effects.

Topical drugs are generally recommended as the first-line treatment for mild acne patients, like retinoic acid, benzoyl peroxide, and topical antibiotics. And for moderate to severe acne, a combination of topical drugs, systemic drugs, and phototherapy is often recommended to achieve better outcomes (4). In recent years, there has been significant progress in the development of novel therapies for acne, targeting various pathogenic mechanisms. These new treatments offer promising results and have generated significant interest. This article aims to review the recent research progress in acne treatment, highlighting the innovative approaches and potential benefits for patients.

## 2 Topical medication

Topical drugs are widely used and effective in patients with mild to moderate acne. Topical antibiotics, retinoids, benzoyl peroxide, azelaic acid and salicylic acid are commonly used in clinical treatment of acne (Table 1). The choice of topical medication may be influenced by various factors, such as the patient’s age, the location and extent of the lesions, the severity of acne, and the patient’s individual preferences. Other considerations include skin type, potential side effects, drug interactions, and adherence to treatment regimens. In order to enhance the simplicity of the acne treatment and promote patient adherence, a variety of fixed-dose combination products available. Commonly used combinations include antibiotics with benzoyl peroxide, antibiotics with retinoic acid, and retinoic acid with benzoyl peroxide (5).

**Table 1:** Summary of traditional medical options for acne.

Classification of drugs	Drugs	Route of administration	Recommended use
Antibiotics	Erythromycin, minocycline	Topical/oral	Single use is not recommended
	Clindamycin, fusidic acid	Topical	Single use is not recommended
	Doxycycline, roxithromycin, azithromycin	Oral	Single use is not recommended
	Penicillin, sulfonamides, cephalosporins	Oral	Not recommended
Benzoyl peroxide	Benzoyl peroxide	Topical	First-line
Retinoids	Adapalene, tretinoin, tazarotene	Topical	Prevent atrophic scarring and pigmentation
	Isotretinoin, viaminate	Oral	Strict contraception is recommended for pregnant women
Azelaic acid	Azelaic acid	Topical	Pigmentation pigmentation
Salicylic acid	Salicylic acid	Topical	Recommended starts with lower concentrations
Hormone therapy	Androgens, estrogens, insulin, insulin-like growth factors	Oral	
	Spironolactone	Oral	Recommended for hormonal acne

### 2.1 Antibiotics

*Propionibacterium acnes* (*P. acnes*) plays a pivotal role in the pathogenesis of acne vulgaris. This bacterium colonizes the sebaceous glands and hair follicles of the skin, and its overgrowth can lead to inflammation and the formation of acne lesions. Topical antibiotics are commonly used in acne treatment as they exhibit both anti-*P. acnes* and anti-inflammatory properties. The topical application of antibiotics directly to the affected area can help reduce the bacterial colonization of the skin and hair follicles by *P. acnes*. The decrease in bacterial load can result in reduced inflammation and subsequently lead to a reduction in acne lesions.

At present, the commonly prescribed antibiotics for acne treatment include clindamycin, erythromycin, minocycline, and fusidic acid. Topical clindamycin can be utilized in combination with benzoyl peroxide and retinoic acids. It is generally well tolerated in a variety of concentrations and preparations such as lotions, foams, and gels.

Clindamycin 1% solution or gel is the preferred topical antibiotic for treating acne, while 2% erythromycin can be used in various topical products. However, due to higher resistance and weaker efficacy compared to clindamycin, it’s recommended as a first-line treatment. To prevent drug resistance, the combination therapy with benzoyl peroxide or retinoic acid is often preferred (6). When used in combination with benzoyl peroxide, it can improve efficacy and reduce the development of resistant bacterial strains, while improving patient compliance with treatment regimens (4).

### 2.2 Benzoyl peroxide

The mechanism of benzoyl peroxide is to decompose into benzoic acid and hydrogen peroxide, and then reduce the concentration of *Propionibacterium acnes* by generating free oxygen radicals. It also has anti-inflammatory and keratolytic properties, and currently there is no drug resistance, so it can be used as the preferred topical medicine for inflammatory acne. Research has demonstrated that benzoyl peroxide formulations at concentrations of 2.5, 5, and 10% exhibit comparable efficacy in the treatment of acne vulgaris. Nonetheless, increased concentrations can potentially elicit irritant dermatitis with adverse reactions like dryness, scaling, and erythema. Consequently, clinicians advise initiating therapy with a low concentration and a limited treatment area for cautious titration (7).

### 2.3 Retinoids

Retinoids, derived from vitamin A, are commonly used to treat acne vulgaris due to their ability to regulate skin cell growth and reduce inflammation. They also help clear sebum from glands, dissolve pimples, and prevent atrophic scarring and pigmentation (8). Topical retinoids such as adapalene, tretinoin, and tazarotene are commonly used to treat acne vulgaris. Adapalene gel at 0.1% is the first non-prescription retinoid approved by the FDA for patients aged 12 and older (9). Topical retinoids can cause dry skin, irritation, and increased serum triglycerides. They also pose a risk of severe birth defects, so monthly pregnancy tests and thorough contraceptive counseling are essential (10–12).

The newly developed combination of adapalene (0.3%) and benzoyl peroxide (2.5%) is safe and effective for treating acne vulgaris in individuals aged 12 years and older. In a 24 weeks study, it showed a higher likelihood of reducing scarring compared to the placebo. Over 48 weeks, it consistently reduced acne and scarring, emphasizing its long-term efficacy (13, 14).

### 2.4 Azelaic acid

Azelaic acid boasts multiple properties, including the dissolving of pimples, antibacterial and mild anti-inflammatory activities, as well as regulating follicular keratinization. Furthermore, its ability to reduce pigmentation by inhibiting abnormal melanocyte activity renders it highly beneficial for individuals with sensitive skin or pigmentation challenges. Categorized as a Category B medication during pregnancy, azelaic acid has been found to have high tolerability and can also be used in lactating women. A recent study found that 15% azelaic acid gel is as effective as 0.1% adapalene gel in treating inflammatory acne in adult women, with sustained therapeutic outcomes (15).

### 2.5 Salicylic acid

Salicylic acid is a mild anti-inflammatory and comedolytic agent used alone or with other medications to manage mild acne vulgaris, especially for patients who cannot tolerate retinoic acid or benzoyl peroxide. It’s available in concentrations from 0.5 to 2%. Treatment starts with lower concentrations and increases as needed, up to two or three times daily. If there are adverse reactions like dryness or redness, the frequency of application can be reduced. Higher concentrations can also be used for chemical peeling of the skin surface (4).

## 3 Oral medication

For patients with moderate to severe acne, the preferred treatment typically involves the use of oral medication, or a combination of oral and topical agents, such as antibiotics, retinoids, or hormonal therapy. This approach is chosen based on the severity of the condition and the patient’s specific needs.

### 3.1 Oral antibiotics

Severe acne cases with inflammation, as well as moderate to severe acne unresponsive to topical treatments, and conditions such as acne fulminans and acne conglobate may require oral antibiotics. Tetracycline antibiotics like doxycycline and minocycline, along with macrolide antibiotics such as erythromycin, roxithromycin, and azithromycin are commonly used for treating these types of acne.

Tetracycline antibiotics are the preferred choice for treating acne due to their strong anti-inflammatory effects, which include inhibiting neutrophil chemotaxis and reducing proinflammatory cytokine production (16). They also help reduce *p. acnes* levels. Other antibiotic classes like penicillin, sulfonamides, and cephalosporins are not recommended for acne treatment unless a patient has contraindications to tetracyclines and macrolides.

To prevent *p. acnes* resistance, it is not recommended to use oral antibiotics alone for acne treatment. They are often used in combination with topical retinoids and benzoyl peroxide. Once improvement is seen, topical agents can be used to maintain the effect. Overuse of antibiotics may lead to resistant strains of bacteria that contribute to acne (17).

### 3.2 Retinoids

Systemic use of retinoid acid can suppress sebum production, hyperkeratosis, and the production of proinflammatory cytokines. Isotretinoin is the only orally administered retinoid approved by the FDA for the treatment of severe nodulocystic and recalcitrant acne, particularly acne that leads to scarring. Furthermore, isotretinoin is the only drug recognized for treating the four pathogenic mechanisms of acne: sebum overproduction, hyperkeratinization, *propionibacterium acnes* colonization, and inflammation. It can be used for patients aged 12 years and older who are not pregnant and suffer from moderate to severe acne (18). The adherence and satisfaction with systemic use of isotretinoin were significantly higher compared to topical treatment and oral antibiotics. Common adverse effects encompass dryness and irritation of the skin and mucosa, myalgia, elevated triglycerides and aminotransferases, as well as severe teratogenic effects. It is recommended to conduct regular monitoring of liver function, serum cholesterol, and triglyceride levels at baseline during the course of treatment. In addition, female patients of fertile age who opt to use isotretinoin must stringently adhere to contraception measures 1 month prior to the commencement of treatment, throughout the duration of treatment, and for 3 months following its conclusion (19). While the adverse reactions of viaminate are similar to isotretinoin, they tend to be relatively less severe (20).

Studies have indicated that isotretinoin may be associated with an increased incidence of mood changes, such as depression and suicidal tendencies. Nevertheless, there is insufficient robust evidence to support this association. Therefore, it is advisable to avoid prescribing isotretinoin to patients who exhibit significant depressive symptoms or have a diagnosis of depression (21).

The initial dose of isotretinoic acid for acne treatment is typically 0.25 to 0.5 mg/kg/d, with a recommended routine dose of 0.5 to 1.0 mg/kg/d and a cumulative dose of 120 to 150 mg/kg, depending on efficacy and patient tolerance. The achievement of the appropriate cumulative dose may also lead to relief of acne. Regular doses of isotretinoin are associated with a decreased frequency of acne recurrence compared to low doses (22).

### 3.3 Hormone therapy

Hormonal therapies commonly comprise combined oral contraceptives (COCs) and anti-androgen drugs. Studies have revealed that various hormones, including androgens, estrogen, insulin, insulin-like growth factors, and others, play a pivotal role in the pathogenesis of acne, with androgens being the most significant endogenous contributor (23).

COCs can serve as a part of a comprehensive acne treatment plan. Women with contraceptive needs or menorrhagia may start using COCs early in the acne treatment process, and for women who do not respond favorably to other medications, COCs can be introduced as a supplementary treatment. Additionally, COCs can be used in combination with other oral drugs such as tetracyclines and spironolactone to achieve optimal therapeutic outcomes (24).

Anti-androgen drugs, such as estrogen, progesterone, spironolactone, and insulin sensitizer, are commonly used in clinical practice to reduce the production of sebaceous lipids and alleviate acne symptoms. Spironolactone, a non-selective aldosterone receptor antagonist, exhibits potent antiandrogenic activity by decreasing testosterone production and competitively inhibiting the binding of testosterone and DHT to androgen receptors in the skin. This ultimately leads to decreased sebum production and improved acne symptoms. Spironolactone is considered a long-term treatment option for hormonal acne with recognized safety and tolerance when used alone or in combination with COCs. Although generally well tolerated, dose-related side effects of spironolactone may include polyuria (29%), irregular menstruation (22%), breast tenderness (17%), breast enlargement, fatigue, headache, and dizziness (25). Due to its potassium-preserving diuretic properties, spironolactone carries the potential for hyperkalemia and hypotension. However, a study of 967 healthy female patients aged 18–45 who administered spironolactone for acne revealed no increased risk of hyperkalemia during treatment. Therefore, routine monitoring of potassium levels is unnecessary for female patients under the age of 45 with no additional comorbidities or symptoms (26).

## 4 Physical therapy

The traditional acne treatments are fraught with several limitations, such as prolonged treatment durations, the emergence of antibiotic resistance, intolerable adverse reactions, and challenges with patient compliance.

In the past decade, laser and light-based interventions have emerged as viable adjunctive or alternative modalities for acne treatment, effectively integrating with traditional drug therapies and other physical modalities. These alternative acne treatments offer distinct advantages, such as abbreviated treatment durations, reduced antibiotic usage to mitigate resistance, and enhanced patient compliance. Physical acne treatments primarily encompass photodynamic therapy (PDT), red and blue light therapy, laser therapy, and photon therapy. These modalities can be effectively combined with oral or topical medications as adjunctive or alternative treatments for acne. Furthermore, they can be utilized to address post-acne sequelae such as scarring and hyperpigmentation (27).

### 4.1 Laser therapy

Lasers demonstrate inhibitory effects on sebaceous gland secretion and possess anti-inflammatory properties, effectively mitigating acne-induced erythema through the application of intense pulsed light (IPL) and pulsed dye laser therapies. IPL diminishes sebaceous gland size and quantity, eliminates *P. acnes*, suppresses the production of tumor necrosis factor-α, and stimulates the upregulation of transforming growth factor-β, all of which contribute to the treatment of inflammatory acne (28, 29). CO2 lasers have demonstrated clinical efficacy in treating photoaging, facial rejuvenation, and acne scars (30).

### 4.2 Photodynamic therapy

Photodynamic Therapy (PDT) is an efficient modality for treating inflammatory acne, as it disrupts the hair follicle sebaceous gland unit and reduces the concentration of *P. acnes*. This therapy is particularly useful for moderate to severe acne cases where systemic medication has failed to produce satisfactory results or is not tolerable (31, 32). The principle of PDT for acne treatment involves the application of a photosensitizer, such as topical 5-aminolevulinic acid, to the acne lesions. This photosensitizer accumulates in the hair follicle sebaceous gland unit and metabolizes to produce photosensitive substances. Upon illumination with a specific wavelength of light, photochemical reactions occur, leading to the generation of reactive oxygen species that disrupt the sebaceous glands. This process effectively controls and improves the appearance of acne lesions by eliminating *P. acnes* and reducing sebum secretion. However, it’s important to note that PDT may be accompanied by side effects such as local erythema, swelling, and other reactions.

### 4.3 Red and blue light

Red light and blue light can be utilized individually or in conjunction for acne phototherapy. Specifically, blue light activates endogenous porphyrins to inhibit the proliferation of *P. acnes*, demonstrating antibacterial and anti-inflammatory properties. Nevertheless, blue light has limited dermal penetration depth. Conversely, red light irradiation alone exhibits tissue-repairing and anti-inflammatory effects with superior tissue penetration ability. The combination of red and blue light for acne treatment can produce synergistic effects, enhancing therapeutic outcomes and often serving as an alternative treatment for moderate inflammatory acne lesions (33, 34).

## 5 Chemical exfoliation

Chemical exfoliation, also known as chemical peeling, is a dermatological procedure that employs various chemical agents to induce controlled epidermal and dermal injury, thereby promoting skin regeneration and remodeling to manage skin lesions. Commonly used peeling agents include alpha hydroxy acids, salicylic acid, phenol, and other compounds. In the context of active acne and acne scars, chemical peeling can effectively modulate sebum production, dissolve comedones (pimples), and exhibit anti-inflammatory and antibacterial properties to mitigate acne lesions. It is frequently utilized as an adjunctive therapy for mild to moderate acne and post-acne pigmentation. In cases of active acne, chemical peeling can augment the efficacy of topical medications or serve as maintenance therapy in conjunction with conventional treatment modalities. Furthermore, it can be used in conjunction with other resurfacing therapies for addressing acne scars (35).

## 6 Diet and skincare advice

Increasing evidence suggests a potential link between a high glycemic index diet and dairy product intake with the development of acne. A randomized controlled trial conducted on 23 male patients aged 15 to 25 years with acne revealed that those who adhered to a low glycemic load (LGL) diet showed significant improvements in acne severity, as well as notable reductions in weight and body mass index (BMI), a significant decrease in the free androgen index, and improved insulin sensitivity at the end of 12 weeks (36). In another randomized controlled trial involving 32 participants aged between 20 and 27, researchers discovered that those who were randomly assigned to follow a low glycemic load (LGL) diet showed statistically significant improvements in acne severity, but there were no changes in weight or BMI (37).

Proper selection and use of topical skincare products are the foundation of treating acne. When selecting a dual-action cleanser, it is recommended to first use an oil-based cleanser, followed by a water-based or surfactant cleanser. Additionally, it may temporarily reside on the skin, minimizing damage to the stratum corneum protein. The correct utilization of moisturizers, cleansers, and sunscreens can significantly decrease the prevalence of both inflammatory and non-inflammatory acne lesions (38).

## 7 New perspectives

Extensive research has been conducted in recent years on acne vulgaris itself, as well as available and potential treatment options. This review delves into various treatment modalities for acne, encompassing topical therapy, systemic therapy, phototherapy, and chemical peeling. However, owing to the escalating resistance of *P. acnes* to existing antibiotics and challenges related to patient adherence, novel frontiers in acne therapeutics are continually under exploration (Table 2).

**Table 2:** Summary of new perspectives for acne treatment.

Drugs	Concentration or dosage form	Route of administration	Recommended use
Trifarotene	50 μg/g cream	Topical	Recommended for acne patients aged 9 and older
	100 μg/g cream	Topical	
Minocycline foam	4% minocycline foam	Topical	Strong antibacterial and anti-inflammatory properties
Clascoterone Cream	1% clascoterone Cream	Topical	Attenuated skin erythema
Topical Meclizine Gel	2% Meclizine gel	Topical	
Ivermectin for topical use	The 1% ivermectin cream	Topical	Improve inflammatory acne
Topical surfactant – oil gel		Topical	Recommended for inflammatory acne vulgaris
Topical nitric oxide and its derivatives	NO4% gel	Topical	
Oral probiotics		Oral	Recommended for mild to moderate acne
New antibiotics and related treatments	Pentobra, Zolav	Oral	
Metformin		Oral	Recommended for patients with acne and polycystic ovary syndrome
Biological agents	Tumor necrosis factor-α inhibitors	Oral	Recommended for severe nodular acne and acne fulminans
	IL-17 inhibitors and IL-23 inhibitors	Oral	Recommended for refractory SAPHO syndrome
Microneedles and microneedle patches	Microneedles	Topical	Recommended for improves scar formation
	microneedle patches		Recommended for post-inflammatory hyperpigmentation

### 7.1 Topical treatment

#### 7.1.1 A new class of topical retinoic acid: trifarotene

Trifarotene is a new topical retinoid for treating acne vulgaris in patients aged 9 and older. It works by activating the retinoic acid receptor gamma, which regulates cellular differentiation and keratinization in the pilosebaceous unit. This helps normalize skin shedding, reduce hyperkeratinization, and alleviate comedone formation and inflammatory lesions associated with acne. Studies have demonstrated that trifarotene 50 μg/g cream was well tolerated and safe for use systemically under maximum conditions in both adults and pediatric ance patients, even those with severe acne. Moreover, Safety analyses for trifarotene up to 100 μg/g did not show local or systemic safety concerns (39). Trofarotene’s unique mechanism of action offers an innovative treatment approach for individuals seeking relief from acne vulgaris (40).

#### 7.1.2 New dosage form: minocycline foam

A new 4% minocycline foam has been approved by the Food and Drug Administration (FDA), which is believed to have strong antibacterial and anti-inflammatory properties similar to other tetracycline antibiotics. Scholars have documented that minocycline foam exhibits notable superiority in both antibacterial and anti-inflammatory actions, demonstrating excellent tolerability. This foam may be beneficial for acne patients who have developed resistance to other treatments. The lipophilic properties of this product facilitate its preferential access to hair follicle sebaceous glands, leading to a skin concentration that is 850 times greater than systemic absorption. Notably, the concentration of minocycline in the blood remains almost undetectable, indicating its selectivity and localized effect on the skin (41). The high skin concentration achieved with this treatment significantly reduces the risk of antimicrobial resistance, allowing it to work locally without eliciting severe systemic adverse reactions commonly associated with orally administered minocycline.

#### 7.1.3 Clascoterone cream

1% clascoterone cream, a topical androgen receptor inhibitor, has demonstrated the ability to compete with dihydrotestosterone for androgen receptor binding in sebaceous glands based on *in vitro* studies. Recently approved by the FDA in August 2020 for the treatment of acne, it represents the first acne medication with a novel mechanism of action since the introduction of isotretinoin in 1982. Its position in treatment is currently unclear, but it has the potential to become a new component of combined acne treatment regimens (42).

#### 7.1.4 Tranexamic acid

Tranexamic acid (TXA) is a synthetic derivative of lysine that inhibits the conversion of plasminogen to plasmin, and consequently reducing blood loss (43). Previously, topical TXA has demonstrated its efficacy in improving post-inflammatory erythema induced by acne. Recently, a randomized controlled trial evaluating the treatment of mild to moderate acne demonstrated that topical administration of 10% TXA significantly reduced the count of inflammatory acne pimples and attenuated skin erythema, resulting in a more uniform skin tone, starting from the fourth week of therapy. The side effects include mild erythema and scaling, which can be prevented and alleviated by applying moisturizer. Consequently, the topical administration of 10% TXA offers a promising adjunctive treatment option for individuals with mild to moderate facial acne (44).

#### 7.1.5 Topical Meclizine gel

Meclizine hydrochloride, an H1 histamine receptor antagonist, is typically prescribed in oral form to address allergic skin and mucous membrane conditions, as well as nausea and vomiting associated with motion sickness. Recently, studies have indicated encouraging outcomes when using this medication for the treatment of acne. A recent double-blind, randomized, placebo-controlled clinical trial involving 60 volunteers over a period of 12 weeks demonstrated promising results for the use of 2% Meclizine gel in the treatment of acne. Specifically, the use of this gel led to a statistically reduction in the Acne Severity Index (ASI) by 20.1% (*p* < 0.001) compared to the placebo group, where only an 8.9% decrease was observed (not statistically significant). These findings indicate the potential value of Meclizine in the treatment of acne (45).

#### 7.1.6 Ivermectin for topical use

The 1% ivermectin cream, which has been approved for treating head lice and rosacea, also holds potential in treating acne. This potential is realized by its capacity to mitigate the lipopolysaccharide-mediated inflammation caused by *p. acnes*. A recent scholarly study has revealed that topical 1% ivermectin cream can improve inflammatory acne (5).

#### 7.1.7 Topical surfactant – oil gel

Surfactant is a surface-active agent with emulsifying and antibacterial properties. A recent scholarly study has shown the beneficial impact of surfactant oil gel on reducing inflammatory acne triggered by *P. acnes* in mice. The study included epidermal morphology and histopathological analysis, which confirmed that surfactant oil gel significantly improved the epidermal swelling and erythema caused by the bacterium. Furthermore, following the application of surfactant oil gel, there was a significant reduction in the colony count of Cutibacterium acnes present within the epidermis. With its demonstrated antibacterial and anti-inflammatory activities, surfactant oil gel exhibits therapeutic effects on inflammatory acne vulgaris induced by *P. acnes*, indicating its potential as an innovative therapeutic agent for inflammatory acne vulgaris (46).

#### 7.1.8 Topical nitric oxide and its derivatives

Nitric oxide (NO) is an endogenous rapid-acting gas that possesses immunomodulatory and antimicrobial activities (47). The immune regulatory pathways play a significant role in the pathogenesis of acne. Scholars have utilized NO-based nanoparticle delivery systems to demonstrate that NO-releasing nanoparticles exhibit remarkable bactericidal activity against *P. acnes*, effectively inhibiting its growth and suppressing the release of cytokines such as TNF-α, IL-1β, IL-6, and IL-8 in human cells (48). In a Phase II randomized controlled trial, scholars discovered that the use of NO4% gel (SB204) led to a significant reduction in acne lesions without any noticeable adverse effects (49). While the precise mechanism of how SB204 reduces both inflammatory and non-inflammatory lesion counts remains unclear, the topical application of nitric oxide-releasing compounds may exert an impact on acne and natural immune regulatory pathways associated with acne vulgaris.

### 7.2 Systemic therapy

#### 7.2.1 Oral probiotics

Extensive *in vitro* and *in vivo* research has consistently shown that probiotics exhibit inhibitory effects on *P. acnes*, suppressing the production of the cytokine IL-8 in both epithelial cells and keratinocytes, while also exhibiting immunomodulatory activity (50). A comparative study examining the efficacy of oral probiotics, oral minocycline, and their combined therapy demonstrated significant improvements in acne lesions across all three treatment groups post-treatment, with the combined therapy exhibiting the most pronounced effect (51). In a randomized controlled trial, 114 patients were given a dietary supplement composed of diverse probiotic strains and plant extracts. The findings indicated that the supplement was both safe and effective in treating individuals with mild to moderate acne, demonstrating excellent tolerability (52).

An Italian single-center interventional study included 30 patients with mild to moderate acne who treated with a biotin-based supplement and three lactic ferments strains, which combined with topical gel products composed of azelaic acid, hydroxypinacolone retinoate, and alpha-hydroxy acid, showed that the severity of acne lesions could be improved through the use of topical products combined with systemic supplements, and confirmed the potential therapeutic effect of probiotic supplements in managing mild to moderate acne (53).

#### 7.2.2 New antibiotics and related treatments

The study identified a new peptide-aminoglycoside compound, Pentobra, with superior antibacterial activity against *P. acnes* compared to tobramycin (54). Additionally, researchers discovered the antibiotic Zolav, which effectively inhibits the growth of *P. acnes* in both *in vitro* and *in vivo* acne models. This offers a promising and cost-effective treatment option for acne (55).

#### 7.2.3 Metformin

Metformin, known primarily as a hypoglycemic agent, has recently been studied as a novel therapeutic agent for treating acne. Analyses of current research studies on metformin have revealed its potential benefits for patients with acne who also have polycystic ovary syndrome (PCOS, potentially serving as a standalone or adjunctive therapy for these patients) (56). Some scholars have also found that metformin, as an adjunctive therapy, can be used in combination with tetracycline and topical benzoyl peroxide to treat moderate to severe acne (57).

### 7.3 Biological agents

In sporadic case reports, the use of biologics has been explored for the treatment of severe acne. Tumor necrosis factor-α inhibitors such as adalimumab and etanercept are considered effective drugs for the treatment of severe nodular acne and acne fulminans. Some scholars have discovered that a 12 months continuous treatment of adalimumab led to a significant reduction in symptoms of acne vulgaris among patients (58). Furthermore, a notable case study involved a 26-year-old male patient who showed significant clinical improvement with a biweekly dose of 40 mg adalimumab. This case provides additional evidence supporting the therapeutic use of adalimumab for acne treatment (59). Researchers have investigated SAPHO syndrome and ascertained that IL-17 inhibitors and IL-23 inhibitors exhibit promising efficacy in the management of refractory SAPHO syndrome, leading to significant improvement in patients’ acne symptoms (60, 61).

### 7.4 Microneedles and microneedle patches

#### 7.4.1 Microneedles

Acne scars can be effectively treated using advanced methods such as dermabrasion, laser therapy, chemical peeling, cryotherapy, steroid injections, fillers, and surgery. However, challenges persist due to adverse events and varying patient responses. For instance, ablative laser therapy is not recommended for patients with darker skin tones due to a higher risk of severe tissue reactions (62). Microneedle devices have also improved scarring by enabling percutaneous administration of topical retinoic acid and salicylic acid (63).

#### 7.4.2 Microneedle patch

A study found that acne lesions and post-inflammatory hyperpigmentation were significantly improved among acne subjects who were treated with daily applications of anti-acne microneedle (AA-DMN) patches for a consecutive 28-day period, without causing adverse skin reactions (64, 65). Microneedle technology improves drug delivery to the lesion area, enhancing treatment outcomes and patient compliance for acne therapy (5).

### 7.5 Optimize patient management

As is widely acknowledged, the most challenging aspect of managing acne lies in enhancing patient compliance and reducing the frequency of recurrence. During the past few years of the COVID-19 outbreak, the frequent mask-wearing has resulted in a rise in the occurrence of facial acne among the general public. This phenomenon is so prevalent that it has given rise to a new descriptive term, maskne, which refers to mechanical acne (66, 67). Some researchers have observed that use teledermatology for acne management has contributed to improved compliance among acne patients and yielded superior treatment outcomes during the COVID-19 pandemic (68, 69). Improve these services and increase the safety of and satisfaction with teledermatology consultations for patients and dermatologists, especially for patients who need maintenance treatment are capable of sustaining their ongoing treatment, Thus enhancement of patient satisfaction and treatment efficacy (70).

## 8 Conclusion

Acne is a multifactorial, chronic inflammatory dermatological condition with a complex pathogenesis. Despite the availability of numerous topical and systemic treatment modalities, a substantial proportion of patients continue to grapple with achieving sustained therapeutic efficacy. Fortunately, ongoing research into the pathogenesis of acne has precipitated the rapid evolution of novel therapeutic strategies. These innovative interventions offer the potential to optimize clinical outcomes, bolster patient adherence, and engender heightened patient satisfaction.

## Author contributions

YL: Writing – original draft, Writing – review & editing, Conceptualization, Data curation, Formal analysis, Investigation, Methodology, Resources, Validation. XH: Writing – review & editing. GD: Writing – review & editing. XW: Writing – review & editing. TL: Writing – review & editing.
